# Supplementary material for: Non-Invasive Blood Flow Speed Measurement Using Optics
Source: Sensors (Basel). 2022 Jan 25;22(3):897. doi: 10.3390/s22030897 (PMC8838687; doi:10.3390/s22030897)
Supplement: Supplementary file 1 [file sensors-22-00897-s001.zip › Supplementary Material.pdf]

# Supplementary Materials: Non-Invasive Blood Flow Speed Measurement Using Optics

**Alex Ce Zhang [1], Yu-Hwa Lo [1,*]**

Starting from the Helmholtz equation:

$$[\nabla^2 + k^2]G_1(\vec{r},\tau) = -\frac{S(\vec{r})}{D} \tag{S1}$$

in which

$$k = jW(\tau) \tag{S2}$$

Later on we will see that the imaginary number would change the regular bessel function to a modified bessel function and it's linear combinations. It's solution will not exhibit oscilation behaviour but will be an exponential function.

The total field would be a linear combination of the incident field and the scattered field, denoted as $G_1^i$ and $G_1^s$. Let's first look at the scattered field.

$$G_1(\vec{r},\tau) = G_1^{in}(\vec{r},\tau) + G_1^{sc}(\vec{r},\tau) \tag{S3}$$

## 1. Scattered Field, Homogeneous Solution

And the scattered field would follow the homogeneous form of equation (S1):

$$[\nabla^2 + k^2]G_1^{sc}(\vec{r},\tau) = 0 \tag{S4}$$

As illustrated in Figure S1, we choose the $x$-axis as the cylinder direction and we assume the cylinder length is infinite. And equation (S4) becomes a 2d helmholtz equation, and can be expressed in polar coordinates(y-z plane) utilizing the symmetry property of the cylinder.

The solution can be obtained using the separation of variable[1]:

$$G_1^{sc} = \psi_1(r)\psi_2(\theta) \tag{S5}$$

And this $r$ is the radial component under the new coordinate system which is a different from the $\vec{r}$ in the previous equations. The laplacian under polar coordinates can be written as:

$$\nabla^2 G_1^{sc} = \frac{1}{r}\frac{\partial}{\partial r}\left(r\frac{\partial G_1^{sc}}{\partial r}\right) + \frac{1}{r^2}\frac{\partial^2 G_1^{sc}}{\partial \theta^2} \tag{S6}$$

Substituting back into equation (S4):

$$\frac{1}{r}\frac{\partial}{\partial r}\left\{r\frac{\partial}{\partial r}[\psi_1(r)\psi_2(\theta)]\right\} + \frac{1}{r^2}\frac{\partial^2[\psi_1(r)\psi_2(\theta)]}{\partial \theta^2} + k^2\psi_1(r)\psi_2(\theta) = 0 \tag{S7}$$

After re-arranging the terms, the above equation can be written as:

$$\frac{r^2}{\psi_1(r)}\frac{d^2\psi_1}{dr^2} + \frac{r}{\psi_1(r)}\frac{d\psi_1}{dr} + k^2r^2 = -\frac{1}{\psi_2(\theta)}\frac{d^2\psi_2(\theta)}{d\theta^2} \tag{S8}$$

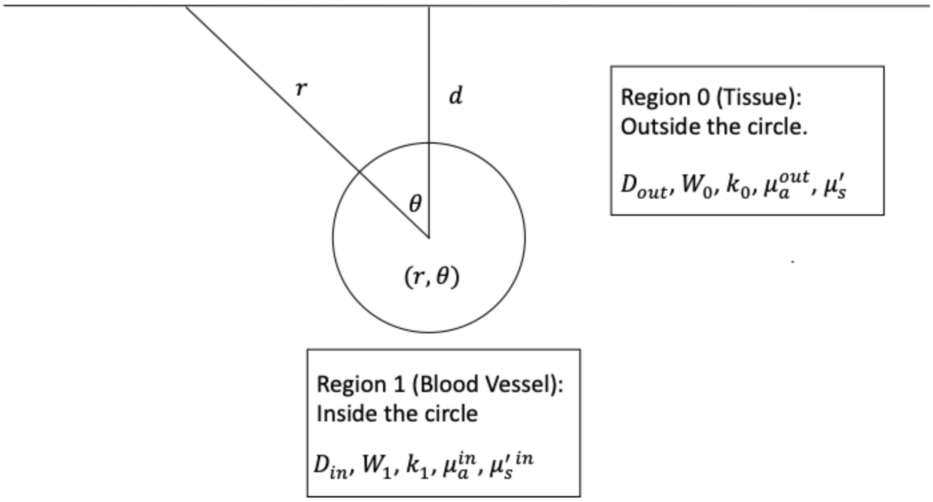


**Figure S1.** This figure is an illustration of the geometry used for numerical calculation using a plane illumination from the top surface. The coordinate system in this figure is polar coordinate, the center $r = 0$ is located at the center of the blood vessel. The region inside the circle represents the blood vessel, the area outside the circle represents the tissue which is semi-infinite.As defined previously, $D = \frac{1}{3\mu_s'}$ is photon diffusivity with unit of meter and $\mu_s'$ is the reduced scattering coefficient, $\mu_a$ is the absorption coefficient with unit of $m^{-1}$ , $k$ and $W$ is wavevector defined in equation (S1) and (S2). Subscript and superscript of "in" and "out" denotes whether it's inside the blood vessel or outside of the blood vessel

Use separation of variable for equation (S8), the LHS is only a function of $r$ and RHS is only a function of $\theta$. Define a constant $m$:

$$\frac{1}{\psi_2(\theta)}\frac{d^2\psi_2(\theta)}{d\theta^2} = -m^2 \tag{S9}$$

$$\frac{r^2}{\psi_1(r)}\frac{d^2\psi_1}{dr^2} + \frac{r}{\psi_1(r)}\frac{d\psi_1}{dr} + k^2r^2 = m^2 \tag{S10}$$

The solution for equation (S9) is:

$$\psi_2(\theta) = e^{jm\theta} \tag{S11}$$

Applying the periodical condition, i.e. $\psi_2(\theta) = \psi_2(\theta + 2\pi)$ . So $m$ will has to be an integer. Equation (S10) can be written as:

$$r^2\frac{d^2\psi_1(r)}{dr^2} + r\frac{d\psi_1(r)}{dr} + (k^2r^2 - m^2)\psi_1(r) = 0 \tag{S12}$$

The solution to the above equation can be written in the form of Bessel functions:

$$\psi_1(r) = A_m J_m(kr) + B_m Y_m(kr) \tag{S13}$$

In which, $J_m$ and $Y_m$ is the Bessel function of the first kind of order $m$ and Bessel function of the second kind of order $m$. And $A_m$, $B_m$ are constants depending on the boundary condition.
Equation (S13) can also be written as the combination of Hankel functions:

$$\psi_1(r) = C_m H_m^{(1)}(kr) + D_m H_m^{(2)}(kr) \tag{S14}$$

in which, the Hankel functions are a combination of Bessel functions:

$$H_m^{(1)}(kr) = J_m(kr) + jY_m(kr) \tag{S15}$$

which is called the Hankel function of the first kind of order $m$.

$$H_m^{(2)}(kr) = J_m(kr) - jY_m(kr) \tag{S16}$$

which is called the Hankel function of the second kind of order $m$. And the constants have the following relation: $A_m = C_m + D_m$, $B_m = j(C_m - D_m)$
Combine the solution and all the possible values of $m$, the scattered field can be written as:

$$G_1^{sc}(r, \theta, \tau) = \sum_{m=-\infty}^{m=+\infty} e^{jm\theta}[A_m J_m(kr) + B_m Y_m(kr)] \tag{S17}$$

Or in the form of Hankel function:

$$G_1^{sc}(r, \theta, \tau) = \sum_{m=-\infty}^{m=+\infty} e^{jm\theta}[C_m H_m^{(1)}(kr) + D_m H_m^{(2)}(kr)] \tag{S18}$$

The above equation can be simplified due to a physical constraint of the scalar wave satisfying helmholtz equation proposed by German Physicist Sommerfeld. It basically means, the scalar wave must radiates its energy to the infinity, not the other way, no energy may radiate coming from infinity which can be mathematically expressed as the Sommerfeld Far field condition[2]:

$$\lim_{r \to \infty} \sqrt{r}\left(\frac{\partial}{\partial r} - jk\right)G_1^{sc} = 0 \tag{S19}$$

The asymptotic expression of the two Hankel functions when the argument $(kr)$ goes to infinity can be written as [3]:

$$H_m^{(1)}(kr) = \sqrt{\frac{2}{\pi kr}} e^{j(kr - \frac{1}{2}m\pi - \frac{\pi}{4})} \tag{S20}$$

$$H_m^{(2)}(kr) = \sqrt{\frac{2}{\pi kr}} e^{-j(kr - \frac{1}{2}m\pi - \frac{\pi}{4})} \tag{S21}$$

Using Sommerfeld condition as in equation (S19), with a simple derivative, we can easily find that the Hankel function of the second kind does not satisfy the Sommerfeld far field condition. In other words, in equation (S18), coefficient $D_m$ has to be zero.

So, the scattered field can be simplified as:

$$G_1^{sc}(r, \theta, \tau) = \sum_{m=-\infty}^{m=+\infty} e^{jm\theta}[C_m H_m^{(1)}(kr)] \tag{S22}$$

Now, since the k inside and outside the cylinder is different: $k_1$ which would represent the moving scatters and $k_0$ which is the static scatters outside the vessel. Let's assume the vessel/cylinder radius is $a$. So $k = k_0$ when $r > a$ and $k = k_1$ when $r < a$.

## 2. Plane Wave Illumination

The plane wave illumination is assuming an uniform illumination of plane wave on top of the surface. As light scatters when the photon enters the medium, the source term would be the un-scattered photon.

We choose the direction of wave propagation as Z-axis. A plane wave can be written as the following form [4] using the Jacobi–Anger expansion:

$$e^{ikr\cos(\theta)} = \sum_{m=-\infty}^{m=+\infty} j^m J_m(kr) e^{jm\theta} \tag{S1}$$

$S(\vec{r})$ will be replaced by $S_0 \exp[jk_t(d-z)] = S_0 \exp(jk_t d)\exp(-jk_t r\cos(\theta))$, in which $k_t = j\mu_t$, in which $\mu_t$ is the total scattering cross section, $d$ is the depth of the vessel from the surface of tissue where the plane wave incident from and $S_0$ is the source intensity.
In order to find the inhomogeneous solution, let

$$\begin{aligned} G_1^{in}(r,\theta) &= AS_0 \exp[jk_t(d-z)] \\ &= AS_0 \exp(-\mu_t d)\exp(\mu_t z) \end{aligned} \tag{S2}$$

And $A$ is unknown, substitute the above equation into the following equation(diffusion equation):

$$[\nabla^2 + k^2]G_1(\vec{r},\tau) = -\frac{S(\vec{r})}{D} \tag{S3}$$

Since it's the diffusion equation for the outside medium, the above equation can be written as:

$$[\nabla^2 - \frac{\mu_a^{out}}{D_{out}}]G_1(\vec{r},\tau) = -\frac{S(\vec{r})}{D_{out}} \tag{S4}$$

$A$ can be found out to be:

$$A = \frac{1}{\mu_a - \mu_t^2 D_{out}} \tag{S5}$$

The inhomogeneous solution can then be written as:

$$\begin{aligned} G_1^{in}(r,\theta) &= \frac{S_0 \exp(-\mu_t d)}{\mu_a - \mu_t^2 D_{out}} \exp(\mu_t z) \\ &= B\exp(\mu_t z) \\ &= B \sum_{m=-\infty}^{m=+\infty} j^m J_m(-k_t r) e^{jm\theta} \end{aligned} \tag{S6}$$

The total solution can be written as:

$$G_{1,out}(r,\theta,\tau) = \sum_{m=-\infty}^{m=+\infty} Bj^m J_m(-k_t r) + C_m H_m^{(1)}(k_0 r)] e^{jm\theta} \tag{S7}$$

For the field inside the vessel/cylinder, we use equation (S17) instead of the Hankel function. Immediately, we can see that $B_m = 0$ as the Bessel function of second kind, $Y_m$ diverges when $r$ approaching 0. So the scattered wave inside the cylinder is:

$$G_{1,in}(r,\theta,\tau) = \sum_{m=-\infty}^{m=+\infty} e^{jm\theta} A_m J_m(k_1 r) \tag{S8}$$

### 2.1. Boundary Condition

The boundary condition requires the photon density or the correlation function to be continuous at the cylinder boundary and also the flux to be continuous normal to the boundary and it can be expressed as follows[5,6]:

$$G_{1,in}(a,\theta,\tau) = G_{1,out}(a,\theta,\tau) \tag{S9}$$

And:

$$D_{in}\frac{\partial G_{1,in}(r,\theta,\tau)}{\partial r}\bigg|_{r=a} = D_{out}\frac{\partial G_{1,out}(r,\theta,\tau)}{\partial r}\bigg|_{r=a} \tag{S10}$$

The above two boundary conditions can be written as:

$$\sum_{m=-\infty}^{m=+\infty} e^{jm\theta} A_m J_m(k_1 a) = \sum_{m=-\infty}^{m=+\infty} [Bj^m J_m(-k_t a) + C_m H_m^{(1)}(k_0 a)] e^{jm\theta} \tag{S11}$$

And,

$$D_{in}k_1 \sum_{m=-\infty}^{m=+\infty} e^{jm\theta} A_m J'_m(k_1 a) = D_{out} \sum_{m=-\infty}^{m=+\infty} [-Bk_t j^m J'_m(-k_t a) + k_0 C_m H_m'^{(1)}(k_0 a)] e^{jm\theta} \tag{S12}$$

The above relation is valid for all $m$ and $\theta$, and thus, it can be reduced to:

$$A_m J_m(k_1 a) = Bj^m J_m(-k_t a) + C_m H_m^{(1)}(k_0 a) \tag{S13}$$

$$D_{in}k_1 A_m J'_m(k_1 a) = -D_{out}Bk_t j^m J'_m(-k_t a) + D_{out}k_0 C_m H_m'^{(1)}(k_0 a) \tag{S14}$$

From the above two equations, $A_m$ and $C_m$ can be solved as:

$$A_m = Bj^m \frac{D_{out}k_0 J_m(-k_t a) H_m'^{(1)}(k_0 a) + D_{out}k_t J'_m(-k_t a) H_m^{(1)}(k_0 a)}{D_{out}k_0 J_m(k_1 a) H_m'^{(1)}(k_0 a) - D_{in}k_1 J'_m(k_1 a) H_m^{(1)}(k_0 a)} \tag{S15}$$

$$C_m = Bj^m \frac{D_{in}k_1 J'_m(k_1 a) J_m(-k_t a) + D_{out}k_t J_m(k_1 a) J'_m(-k_t a)}{D_{out}k_0 J_m(k_1 a) H_m'^{(1)}(k_0 a) - D_{in}k_1 J'_m(k_1 a) H_m^{(1)}(k_0 a)} \tag{S16}$$

For our interest, since the term of the incident field is not $\tau$ dependent, we can only look at the the scattered field outside the cylinder:

$$G_{1,out}^s(r,\theta,\tau) = \sum_{m=-\infty}^{m=+\infty} C_m H_m^{(1)}(k_0 r) e^{jm\theta} \tag{S17}$$

Substitute in $C_m$:

$$G_{1,out}^s(r,\theta,\tau) = \sum_{m=-\infty}^{m=+\infty} Bj^m \frac{D_{in}k_1 J'_m(k_1 a) J_m(-k_t a) + D_{out}k_t J_m(k_1 a) J'_m(-k_t a)}{D_{out}k_0 J_m(k_1 a) H_m'^{(1)}(k_0 a) - D_{in}k_1 J'_m(k_1 a) H_m^{(1)}(k_0 a)} H_m^{(1)}(k_0 r) e^{jm\theta} \tag{S18}$$

### 2.2. Solution Simplification

The above equation can be simplified using the following recurrence relation:

$$f_{-n}(x) = (-1)^n f_n(x) \tag{S19}$$

Rewrite equation (S7):

$$\begin{aligned}G^{s}_{1,out}(r,\theta,\tau) &= \sum_{m=-\infty}^{m=+\infty} Bj^{m}\frac{D_{in}k_1J'_m(k_1a)J_m(-k_ta)+D_{out}k_tJ_m(k_1a)J'_m(-k_ta)}{D_{out}k_0J_m(k_1a)H'^{(1)}_m(k_0a)-D_{in}k_1J'_m(k_1a)H^{(1)}_m(k_0a)}H^{(1)}_m(k_0r)e^{jm\theta}\\ &= B\frac{D_{in}k_1J'_0(k_1a)J_0(-k_ta)+D_{out}k_tJ_0(k_1a)J'_0(-k_ta)}{D_{out}k_0J_0(k_1a)H'^{(1)}_0(k_0a)-D_{in}k_1J'_0(k_1a)H^{(1)}_0(k_0a)}H^{(1)}_0(k_0r)\\ &+B\sum_{m=1}^{m=+\infty} j^{m}\frac{D_{in}k_1J'_m(k_1a)J_m(-k_ta)+D_{out}k_tJ_m(k_1a)J'_m(-k_ta)}{D_{out}k_0J_m(k_1a)H'^{(1)}_m(k_0a)-D_{in}k_1J'_m(k_1a)H^{(1)}_m(k_0a)}H^{(1)}_m(k_0r)\\ &[e^{jm\theta}+(-1)^m e^{-jm\theta}]\end{aligned} \tag{S20}$$

The derivative can be replaced by the reccurence relation as follows:

$$f'_m(x)=\frac{m}{x}f_m(x)-f_{m+1}(x) \tag{S21}$$

In which, the function $f$ could be $J_m(x)$, $H^{(1)}_m(x)$

In particular, when $m=0$:

$$f'_0(x)=-f_1(x) \tag{S22}$$

equation (S20) can then be written as:

$$\begin{aligned}\frac{G^{s}_{1,out}(r,\theta,\tau)}{B} &= \frac{D_{in}k_1J'_0(k_1a)J_0(-k_ta)+D_{out}k_tJ_0(k_1a)J'_0(-k_ta)}{D_{out}k_0J_0(k_1a)H'^{(1)}_0(k_0a)-D_{in}k_1J'_0(k_1a)H^{(1)}_0(k_0a)}H^{(1)}_0(k_0r)\\ &+\sum_{m=1}^{m=+\infty} j^{m}\frac{D_{in}k_1J'_m(k_1a)J_m(-k_ta)+D_{out}k_tJ_m(k_1a)J'_m(-k_ta)}{D_{out}k_0J_m(k_1a)H'^{(1)}_m(k_0a)-D_{in}k_1J'_m(k_1a)H^{(1)}_m(k_0a)}H^{(1)}_m(k_0r)\\ &[e^{jm\theta}+(-1)^m e^{-jm\theta}]\\ &=\frac{-D_{in}k_1J_1(k_1a)J_0(-k_ta)-D_{out}k_tJ_0(k_1a)J_1(-k_ta)}{-D_{out}k_0J_0(k_1a)H^{(1)}_1(k_0a)+D_{in}k_1J_1(k_1a)H^{(1)}_0(k_0a)}H^{(1)}_0(k_0r)\\ &+\sum_{m=1}^{m=+\infty} j^{m}\frac{D_{in}k_1[\frac{m}{k_1a}J_m(k_1a)-J_{m+1}(k_1a)]J_m(k_0a)+D_{out}k_tJ_m(k_1a)[\frac{m}{-k_ta}J_m(-k_ta)-J_{m+1}(-k_ta)]}{D_{out}k_0J_m(k_1a)[\frac{m}{k_0a}H^{(1)}_m(k_0a)-H^{(1)}_{m+1}(k_0a)]-D_{in}k_1[\frac{m}{k_1a}J_m(k_1a)-J_{m+1}(k_1a)]H^{(1)}_m(k_0a)}\\ &H^{(1)}_m(k_0r)[e^{jm\theta}+(-1)^m e^{-jm\theta}]\end{aligned} \tag{S23}$$

From the above equation, we can see that only the zeroth order and even order will be non-zero. Also, due to the small value of the argument, after computing the remaining even series, the 2nd order will already be 5-6 order magnitude lower than the zeroth order. So In the remaining part, we will only retain the zeroth order.
$k_0$ and $k_1$ are pure imaginary, and $k_0=jW_0$, and $k_1=jW_1(\tau)$, we will need the following modified bessel function which would show no oscillations. Again, I changed the upper case K to W to avoid confusion between modified bessel function of the second kind.

$$J_m(jx)=j^mI_m(x) \tag{S24}$$

and:

$$H_m^{(1)}(jx) = \frac{2}{\pi} j^{-m-1} K_m(x) \tag{S25}$$

The zeroth order term can be then written as:

$$\begin{aligned} & \frac{D_{in} k_1 J_0'(k_1 a) J_0(-k_t a) + D_{out} k_t J_0(k_1 a) J_0'(-k_t a)}{D_{out} k_0 J_0(k_1 a) H_0'^{(1)}(k_0 a) - D_{in} k_1 J_0'(k_1 a) H_0^{(1)}(k_0 a)} H_0^{(1)}(k_0 r) \\ = & \frac{-D_{in} k_1 J_1(k_1 a) J_0(-k_t a) - D_{out} k_t J_0(k_1 a) J_1(-k_t a)}{-D_{out} k_0 J_0(k_1 a) H_1^{(1)}(k_0 a) + D_{in} k_1 J_1(k_1 a) H_0^{(1)}(k_0 a)} H_0^{(1)}(k_0 r) \\ = & \frac{D_{in} W_1 I_1(W_1 a) I_0(-\mu_t a) + D_{out} \mu_t I_0(W_1 a) I_1(-\mu_t a)}{-D_{out} W_0 I_0(W_1 a) K_1(W_0 a) - D_{in} W_1 I_1(W_1 a) K_0(W_0 a)} K_0(W_0 r) \end{aligned} \tag{S26}$$

So the scattered correlation function containing only the zeroth order will be:

$$G_{1,out}^s(r,\theta,\tau) = B \frac{D_{in} W_1 I_1(W_1 a) I_0(-\mu_t a) + D_{out} \mu_t I_0(W_1 a) I_1(-\mu_t a)}{-D_{out} W_0 I_0(W_1 a) K_1(W_0 a) - D_{in} W_1 I_1(W_1 a) K_0(W_0 a)} K_0(W_0 r) \tag{S27}$$

The total measured field will be:

$$G_{1,out}(r,\theta,\tau) = B \exp(\mu_t z) + B \frac{D_{in} W_1 I_1(W_1 a) I_0(-\mu_t a) + D_{out} \mu_t I_0(W_1 a) I_1(-\mu_t a)}{-D_{out} W_0 I_0(W_1 a) K_1(W_0 a) - D_{in} W_1 I_1(W_1 a) K_0(W_0 a)} K_0(W_0 r) \tag{S28}$$

We can make one approximation to simplify the above equation, in the numerator, typically, the first term will be order magnitude larger than the second term. Therefore, in order to understand the underlining physics better, we will drop the second term and the above equation becomes:

$$\begin{aligned} G_{1,out}(r,\theta,\tau) &= B \exp(\mu_t z) + B \frac{D_{in} W_1 I_1(W_1 a) I_0(-\mu_t a)}{-D_{out} W_0 I_0(W_1 a) K_1(W_0 a) - D_{in} W_1 I_1(W_1 a) K_0(W_0 a)} K_0(W_0 r) \\ &= B \exp(\mu_t z) + B \frac{D_{in} I_0(-\mu_t a)}{-D_{out} \frac{W_0 I_0(W_1 a)}{W_1 I_1(W_1 a)} K_1(W_0 a) - D_{in} K_0(W_0 a)} K_0(W_0 r) \\ &\approx B \exp(\mu_t z) - B \frac{D_{in} I_0(-\mu_t a)}{D_{out} \frac{1}{W_1 a} + D_{in} K_0(W_0 a)} K_0(W_0 r) \\ &= \frac{S_0}{\mu_a - \mu_t^2 D_{out}} \left[ 1 - \frac{\exp(-\mu_t d) D_{in} I_0(-\mu_t a)}{D_{out} \frac{1}{W_1 a} + D_{in} K_0(W_0 a)} K_0(W_0 r) \right] \end{aligned} \tag{S29}$$

In which,

$$W_1(\tau) = \sqrt{\frac{1}{D_{in}} [\mu_a^{in} + \frac{1}{3} \mu_s' k_\lambda^2 \langle \Delta r^2(\tau) \rangle]} \tag{S30}$$

$$W_0 = \sqrt{\frac{\mu_a^{out}}{D_{out}}} \tag{S31}$$

For motion caused by convective flow, the above equation could be rewritten as:

$$W_1(\tau) = \sqrt{\frac{1}{D_{in}} [\mu_a^{in} + \frac{1}{3} \mu_s' k_\lambda^2 V^2 \tau^2]} = \sqrt{c + b\tau^2} \tag{S32}$$

Define the following $\tau$ independent function to simplify the equation

$$f_1 = \exp(-\mu_t d) D_{in} I_0(-\mu_t a) K_0(W_0 r) \tag{S33}$$

$$f_2 = D_{in} K_0(W_0 a) \tag{S34}$$

Then, the normalized correlation function can be written as:

$$g_1(r,\theta,\tau) = \frac{G_{1,out}(r,\theta,\tau)}{G_{1,out}(r,\theta,0)} = \frac{1 - \frac{f_1}{\frac{D_{out}}{W_1(\tau)a} + f_2}}{1 - \frac{f_1}{\frac{D_{out}}{W_1(\tau=0)a} + f_2}} \tag{S35}$$

The above equation can be written in the form of:

$$g_1(r,a,\theta,\tau) = \frac{1 - g_1(r,a,\theta,\infty)}{1 + \tau/T} + g_1(r,a,\theta,\infty) \tag{S36}$$

It can be found out that:

$$g_1(r,a,\theta,\infty) = \left[1 - \frac{f_1}{f_2}\right]\left[1 - \frac{f_1}{\frac{D_{out}}{a\sqrt{\frac{\mu_a^{in}}{D_{in}}}} + f_2}\right]^{-1} \tag{S37}$$

$$\begin{aligned} T_F &= \frac{\tau(a\sqrt{c} f_2 + D_{out})}{a f_2(\sqrt{b\tau^2 + c} - \sqrt{c})} \\ &\approx \frac{(a\sqrt{c} f_2 + D_{out})}{a f_2 \sqrt{b}} \\ &\approx \sqrt{\frac{c}{b}} \\ &= \frac{1}{V}\sqrt{\frac{3\mu_a^{in}}{\mu_s^{in} k_\lambda^2}} \end{aligned} \tag{S38}$$

$$\frac{1}{T_F} \approx V k_\lambda \sqrt{\frac{\mu_s^{in}}{3\mu_a^{in}}} \tag{S39}$$

And $k_\lambda$ is the wave vector value at wavelength $\lambda$.

## References

1. Morse, P.M.; Feshbach, H. Methods of theoretical physics. *Am. J. Phys.* **1954**, *22*, 410–413.
2. Sommerfeld, A. *Partial differential equations in physics*; Academic press: Cambridge, MA, USA, 1949.
3. Abramowitz, M.; Stegun, I.A.; Romer, R.H. *Handbook of mathematical functions with formulas, graphs, and mathematical tables*; Dover Publication: Mineola, NY, USA, 1988.
4. Colton, D.; Kress, R. *Inverse acoustic and electromagnetic scattering theory*; Springer: New York, NY, USA, 2019; Volume 93.
5. Boas, D.; O'leary, M.; Chance, B.; Yodh, A. Scattering of diffuse photon density waves by spherical inhomogeneities within turbid media: analytic solution and applications. *Proc. Natl. Acad. Sci.* **1994**, *91*, 4887–4891.
6. Bourlier, C.; Pinel, N.; Kubické, G. *Method of moments for 2D scattering problems: basic concepts and applications*; John Wiley & Sons: Hoboken, NJ, USA, 2013.
